# Supplementary material for: Biomimetic Material for Quantification of Methotrexate Using Sensor Based on Molecularly Imprinted Polypyrrole Film and MWCNT/GCE
Source: Biomimetics (Basel). 2023 Feb 12;8(1):77. doi: 10.3390/biomimetics8010077 (PMC9944472; doi:10.3390/biomimetics8010077)
Supplement: Supplementary file 1 [file biomimetics-08-00077-s001.zip › biomimetics-2111220-supplementary.pdf]

Biomimetic material for quantification of methotrexate using sensor based on molecularly imprinted polypyrrole film and MWCNT/GCE

Eduardo Jara[a], Sabir Khan[a,b,d], Jaime Vega-Chacón[a], Ademar Wong[b,c], Lariel Chagas da Silva Neres[b], Gino Picasso[a,#], Maria D.P.T. Sotomayor[b,c,*]

[a]Laboratory of Physical Chemistry Research, Faculty of Sciences, National University of Engineering, Av. Tupac Amaru 210, Peru

[b]Institute of Chemistry, São Paulo State University (UNESP), 14801-970, Araraquara, SP, Brazil

[c]National Institute of Alternative Technologies for Detection, Toxicological Evaluation and Removal of Micropollutants and Radioactive Agents (INCT-DATREM), Araraquara, SP, Brazil

[d]Department of Natural Sciences, Mathematics, and Statistics, Federal Rural University of the Semi-Arid, Mossoró 59625-900, Brazil

*corresponding authors: #gpicasso@uni.edu.pe; *m.sotomayor@unesp.br*

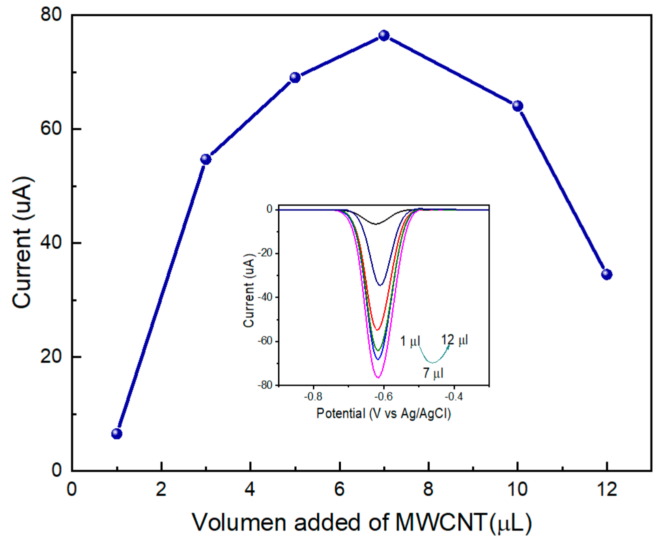

Figure S1. Deposited volume of 1mg/ml MWCNT.

Figure S2 shows the electrochemical profile of methotrexate in not modified electrodes. It can be observed that four peaks were found. The oxidation peak (1) could not be used in the detection, since it could oxidize the polypyrrole polymer that would affect the stability of the sensor. Peak (4) is another methotrexate oxidation peak; however, it only appears if methotrexate reduction has been performed. peak (3), therefore, remains as options to peaks (2) and (3), peak (2) is not stable due to the formation of intermediate species under this potential, for this reason it is not used for a detection method. Peak (3) is a reduction peak and is stable without affecting the sensor or the analyte, so this potential is used for the detection method.

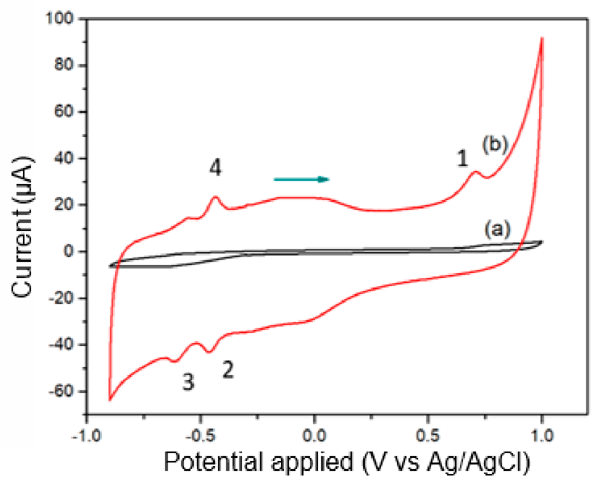

Figure S2. Cyclic voltammetry for (a) GCE and (b) MWCNT/GCE in the standard solution of methotrexate 100 μmol L^{-1}, $\upsilon = 50$ mVs^{-1}.

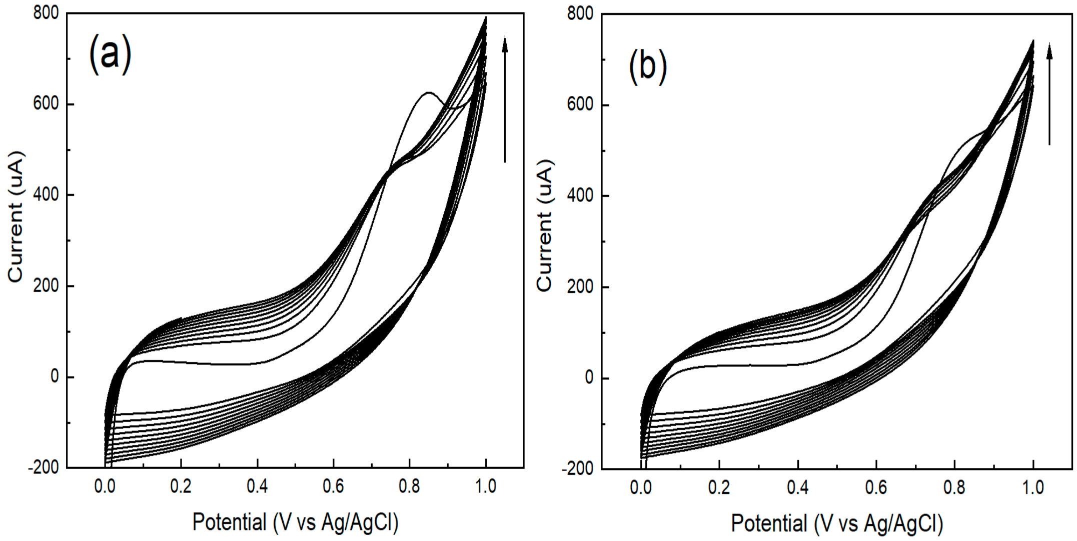

Figure S3. Electropolymerization of 20 mmol L^{-1} pyrrole solution, 0.1 mol L^{-1} $LiClO_4$, and 100 umol L^{-1} methotrexate on the surface of MWCNT/GCE: (a) MIP/MWCNT and (b) NIP/MWCNT.

In Figure S4, can be evidenced that the application of negative or positive potentials during the pre-treatment produces a better repeatability in the pyrrole oxidation, therefore this pre-treatment could favor the orderly growth of the polypyrrole. In other research, it is shown that polypyrrole has a characteristic morphology called "cauliflower", this morphology is due to polymeric growth in the α and β position. Therefore, in the case of applying a potential as a pre-treatment, it can be expected that the morphology will change, since the nucleation is being oriented and a horizontal or vertical growth can occur depending on the applied potential. In this work, a potential of 0.4V was chosen for the pretreatment in order to obtain a vertical growth polymer, as shown in the SEM figure of this manuscript.

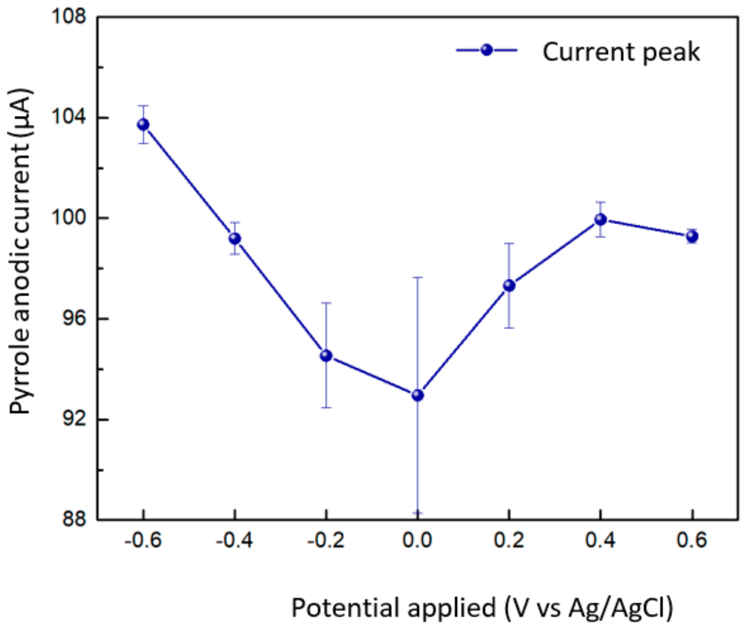

Figure S4. Influence of conditioning potential prior to the electropolymerization of pyrrole.

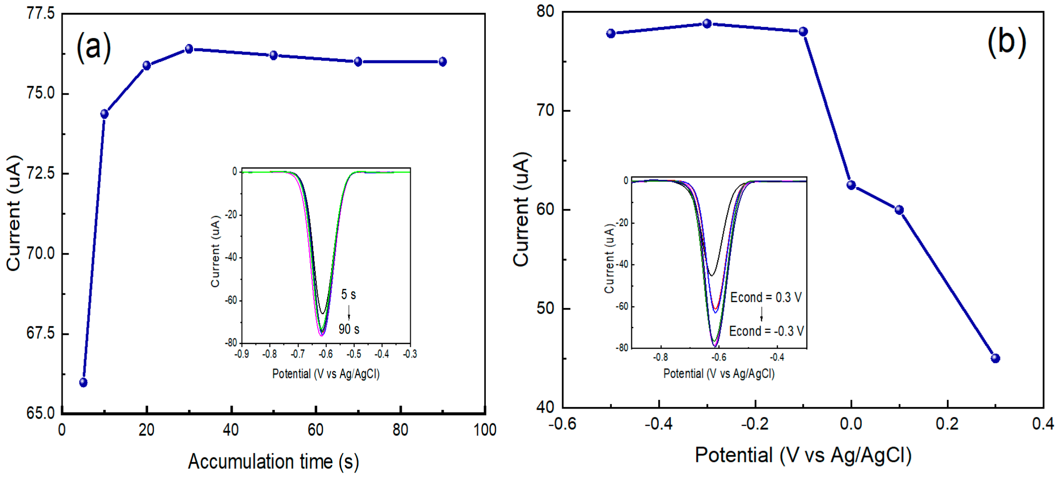

Figure S5. Conditioning parameters: (a) accumulation time and (b) applied accumulation potential.

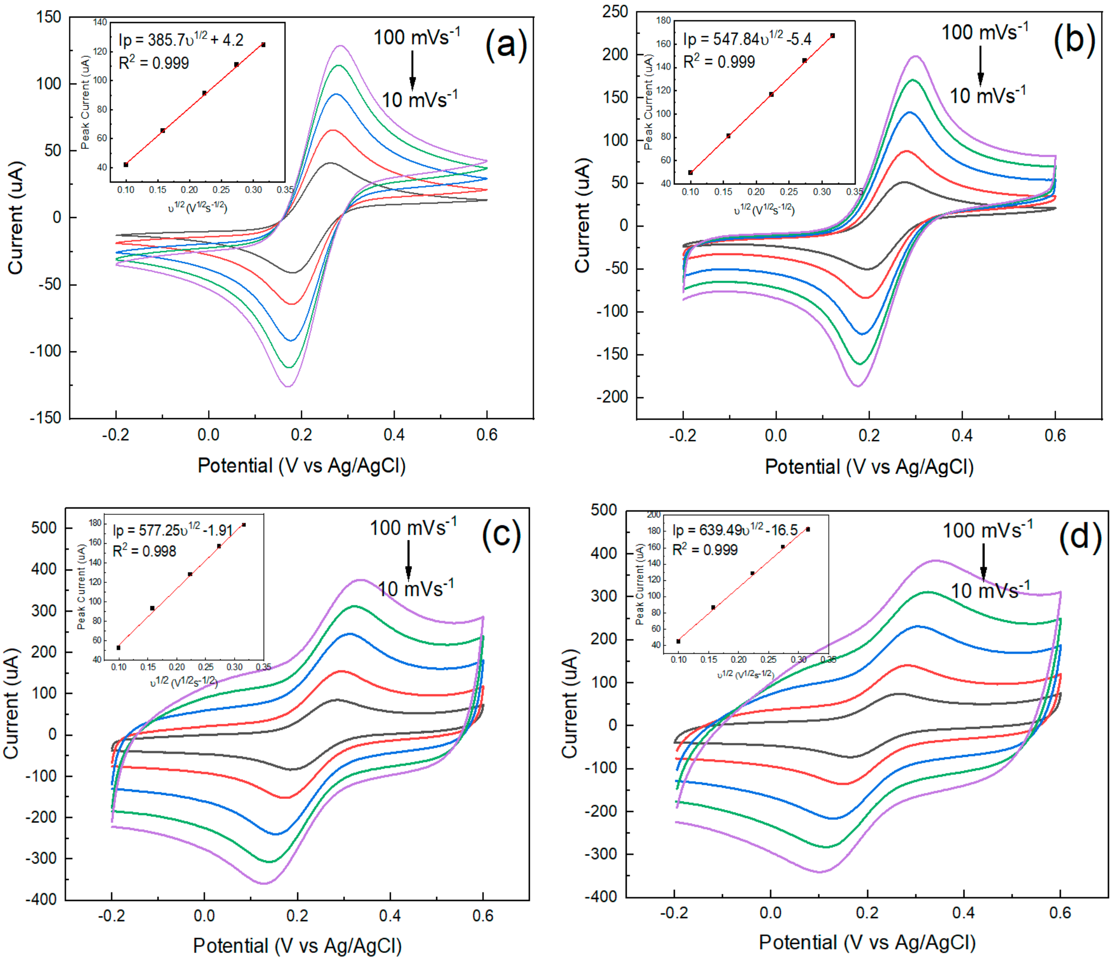

Figure S6. Cycic voltammograms obtained from the application of (a) GCE, (b) MWCNT/GCE, (c) NIP/MWCNT/GCE, and (d) MIP/MWCNT/GCE at the following scan rates:10, 25, 50, 75, and 100 mVs^{-1}.

Figure S7 shows like the pH affect the structure of the MTX. Through this mechanism it is possible to verify why the pH was adjusted up to pH 3 to carry out the detection of this drug and consequently to obtain the optimized conditions for the electrochemical detection.

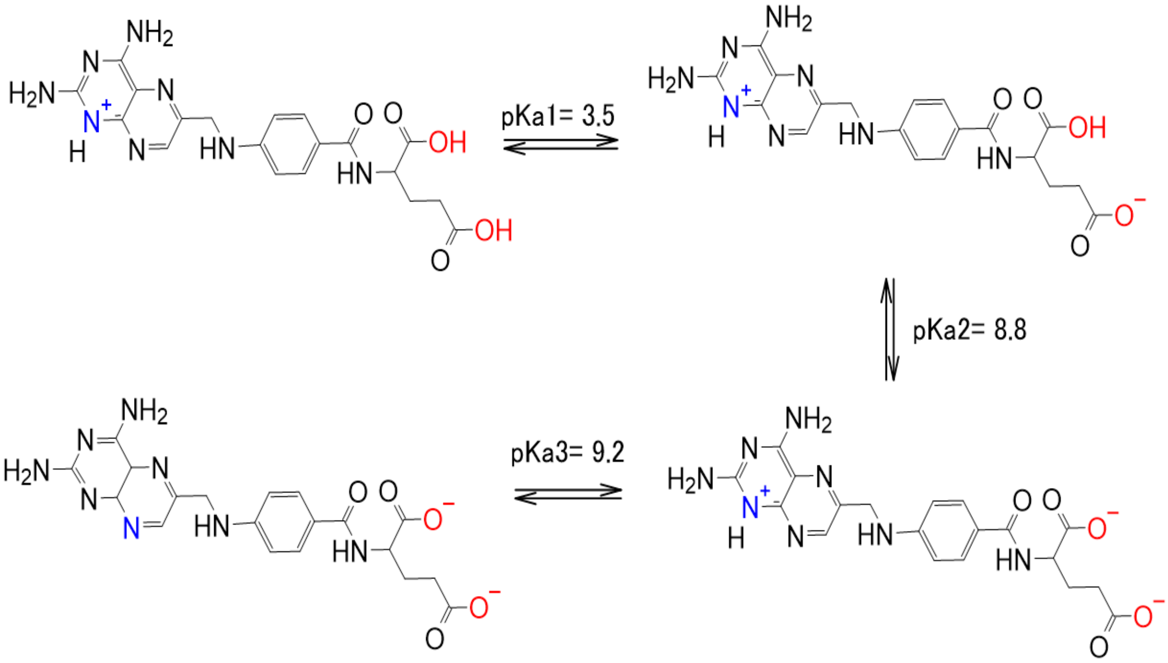

Figure S7. Mechanismo of the acid-base balance in an aqueous methotrexate solution.

Table S1. Summary of detection methods for methotrexate.

Method/sensor	Analyte*	Analytical measure	LOD (mol L^{-1})	Reference
**Graphene Oxide/Polypyrrole**	MTX	UV-visible	2.2×10^{-11}	[S1]
**QD CdTe – MIP**	MTX<br>FA	Fluorescence	3.2×10^{-8}<br>3.4×10^{-8}	[S2]
**HPLC- HRMS**	MTX	-----	4.5×10^{-11}	[S3]
**LC-MS/MS**	MTX	----	2.2×10^{-11}	[S4]
**Poly (l-Lysine)/ GCE**	MTX	SWV	1.7×10^{-9}	[S5]
**f-CNT / GCE**	MTX	DPV	5×10^{-7}	[S6]
**Boron-Doped Diamond Electrode**	MTX	DPV	1×10^{-8}	[S7]
**MIP/MWCNT/GCE**	MTX	DPV	2.7×10^{-9}	This work

*Analyte: MTX = methotrexate; QD CdTe – MIP: molecularly imprinted polymers on dual-color CdTe quantum dots; GCE: Glassy carbon electrode; FA: Folic acid; HPLC: high performance liquid chromatography; HRMS: high resolution mass spectrometry; SWV: Square wave voltammetry; f-CNT: functionalized carbon nanotube; DPV: differential pulse voltammetry.

References:

[S1] Hamidi, Samin, Ayda Azami, and Elnaz Mehdizadeh Aghdam. “A Novel Mixed Hemimicelles Dispersive Micro-Solid Phase Extraction Using Ionic Liquid Functionalized Magnetic Graphene Oxide/Polypyrrole for Extraction and Pre-Concentration of Methotrexate from Urine Samples Followed by the Spectrophotometric Method.” Clinica Chimica Acta 488 (2018) 179–88. https://doi.org/10.1016/j.cca.2018.11.006.

[S2] Ali A. Ensafi, Parisa Nasr-Esfahani, B. Rezaei. “Simultaneous detection of folic acid and methotrexate by an optical sensor based on molecularly imprinted polymers on dual-color CdTe quantum dots”. Analytica Chimica Acta 996 (2017) 64-73. https://doi.org/10.1016/j.aca.2017.10.011

[S3] Dal Bello, Federica, Valentina Santoro, Valentina Scarpino, Chiara Martano, Riccardo Aigotti, Alberta Chiappa, Enrico Davoli, and Claudio Medana. “Antineoplastic Drugs Determination by HPLC-HRMSn to Monitor Occupational Exposure.” Drug Testing and Analysis 8 (2016): 730–37. https://doi.org/10.1002/dta.1827.

[S4] Canal-Raffin, Mireille, Karim Khennoufa, Béatrice Martinez, Yves Goujon, Celia Folch, Dominique Ducint, Karine Titier, Patrick Brochard, Catherine Verdun-Esquer, and Mathieu Molimard. “Highly Sensitive LC–MS/MS Methods for Urinary Biological Monitoring of Occupational Exposure to Cyclophosphamide, Ifosfamide, and Methotrexate Antineoplastic Drugs and Routine Application.” Journal of Chromatography B: Analytical Technologies in the Biomedical and Life Sciences 1038 (2016): 109–117. https://doi.org/10.1016/j.jchromb.2016.10.021.

[S5] Wei, Youli, Liqiang Luo, Yaping Ding, Xiaojing Si, and Yanqun Ning. “Highly Sensitive Determination of Methotrexate at Poly (l-Lysine) Modified Electrode in the Presence of Sodium Dodecyl Benzene Sulfonate.” Bioelectrochemistry 98 (2014): 70–75. https://doi.org/10.1016/j.bioelechem.2014.03.005.

[S6] Kummari, Shekher, V. Sunil Kumar, M. Satyanarayana, and K. Vengatajalabathy Gobi. “Direct Electrochemical Determination of Methotrexate Using Functionalized

Carbon Nanotube Paste Electrode as Biosensor for In-Vitro Analysis of Urine and Dilute Serum Samples." Microchemical Journal 148 (2019): 626–33. https://doi.org/10.1016/j.microc.2019.05.054.

[S7] Šelešovská, Renáta, Lenka Janíková-Bandžuchová, and Jaromíra Chýlková. "Sensitive Voltammetric Sensor Based on Boron-Doped Diamond Electrode for Determination of the Chemotherapeutic Drug Methotrexate in Pharmaceutical and Biological Samples." Electroanalysis 27 (2015): 42–51. https://doi.org/10.1002/elan.201400326.
